# Genomic differences between pure ductal carcinoma *in situ* and synchronous ductal carcinoma *in situ* with invasive breast cancer

**DOI:** 10.18632/oncotarget.3162

**Published:** 2015-03-26

**Authors:** Shinn Young Kim, Seung-Hyun Jung, Min Sung Kim, In-Pyo Baek, Sung Hak Lee, Tae-Min Kim, Yeun-Jun Chung, Sug Hyung Lee

**Affiliations:** ^1^ Department of Microbiology, The Catholic University of Korea, Seoul; ^2^ Department of Pathology, The Catholic University of Korea, Seoul; ^3^ Department of Integrated Research Center for Genome Polymorphism, The Catholic University of Korea, Seoul; ^4^ Department of Hospital Pathology, The Catholic University of Korea, Seoul; ^5^ Department of Medical Informatics, College of Medicine, The Catholic University of Korea, Seoul

**Keywords:** breast cancer, ductal carcinoma *in situ*, genomic difference, whole exome, copy number alteration

## Abstract

Although ductal carcinoma *in situ* (DCIS) precedes invasive ductal carcinoma (IDC), the related genomic alterations remain unknown. To identify the genomic landscape of DCIS and better understand the mechanisms behind progression to IDC, we performed whole-exome sequencing and copy number profiling for six cases of pure DCIS and five pairs of synchronous DCIS and IDC. Pure DCIS harbored well-known mutations (e.g., *TP53, PIK3CA* and *AKT1*), copy number alterations (CNAs) and chromothripses, but had significantly fewer driver genes and co-occurrence of mutation/CNAs than synchronous DCIS-IDC. We found neither recurrent nor significantly mutated genes with synchronous DCIS-IDC compared to pure DCIS, indicating that there may not be a single determinant for pure DCIS progression to IDC. Of note, synchronous DCIS genomes were closer to IDC than pure DCIS. Among the clinicopathologic parameters, progesterone receptor (PR)-negative status was associated with increased mutations, CNAs, co-occurrence of mutations/CNAs and driver mutations. Our results indicate that although pure DCIS has already acquired some drivers, more changes are needed to progress to IDC. In addition, IDC-associated DCIS is more aggressive than pure DCIS at genomic level and should really be considered IDC. Finally, the data suggest that PR-negativity could be used to predict aggressive breast cancer genotypes.

## INTRODUCTION

Breast cancer, a leading cause of cancer-related deaths in women worldwide, represents a genomic disorder in which various types of genomic alterations contribute to initiation and progression of the disease [1]. Mammary ductal carcinoma, the most common type of breast cancer, is largely divided into invasive (invasive ductal carcinoma, IDC) and non-invasive (mainly ductal carcinoma *in situ*, DCIS) tumors. DCIS cells have the morphology of tumor cells, but are still confined to the ducts, while IDC cells penetrate the ducts and exist in the stroma [2].

DCIS is widely accepted as a precursor of IDC [2] and efforts to search for factors that “trigger” invasion are still underway. In colon cancers, genetic alterations are considered the “triggers” for progression of early lesions [3], but it remains uncertain whether DCIS progression to IDC is similar and what genetic alterations are the main triggers. Genetically, DCIS and IDC share gene expression profiles and copy number alterations (CNAs) in common [4, 5]. DCIS and matched adjacent IDC (synchronous DCIS and IDC) have remarkably similar copy number profile [6]. CNAs of synchronous DCIS with IDC are closer to IDC than pure DCIS without IDC [7]. Collectively, these findings suggest that IDCs might develop through genetic evolution from DCIS.

Whole-exome or whole-genome sequencing analysis of IDC [8–10] has found recurrent mutations, including *TP53, PIK3CA, AKT1, GATA3* and *MAP3K1*. Another whole-exome study included pure DCIS, but did not identify any genomic differences between pure DCIS and IDC [10]. A better way to find genetic differences between IDC and DCIS would be to examine three different lesions (pure DCIS devoid of IDC components, synchronous DCIS and IDC). Such an approach would help identify not only the differences between pure DCIS and synchronous DCIS with IDC, but also genomic drivers for the progression of DCIS to IDC. The challenge is separating DCIS and IDC cells in fresh tissues, because DCIS lesions are very small and located very close to the IDC cells.

Here, we attempted to find genomic aberrations that may contribute to the progression of DCIS to invasive diseases by comparing the genomes of pure DCIS, and synchronous DCIS and IDC with whole-exome sequencing and array-comparative genomic hybridization (a-CGH) using microdissection of frozen sections. We found a high genomic concordance of synchronous DCIS and IDC and that pure DCIS displayed fewer driver events than synchronous DCIS with IDC.

## RESULTS

### Whole-exome sequencing profiles

**Figure 1:**
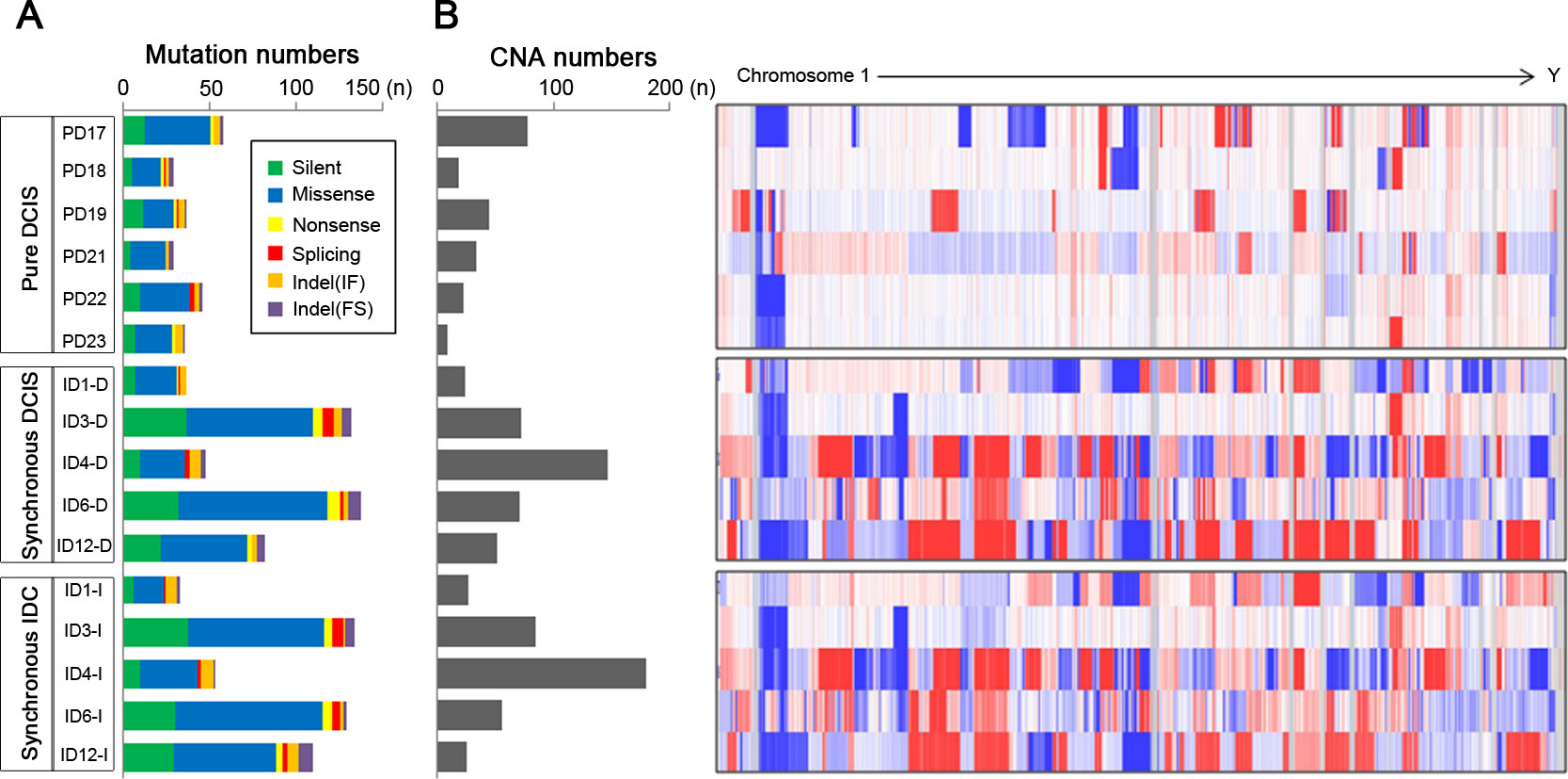
Abundance of somatic mutations and copy number alterations (CNAs) in 6 pure DCIS, 5 synchronous DCIS and 5 synchronous IDC genomes **(A)** The numbers of somatic mutations are shown for the 6 pure DCIS (top) (PD17, PD18, PD19, PD21, PD22 and PD23), 5 synchronous DCIS (middle) (ID1-D, ID3-D, ID4-D, ID6-D and ID12-D) and 5 synchronous IDC (bottom) (ID1-I, ID3-I, ID4-I, ID6-I and ID12-I) genomes with respect to the 6 categories (insets). **(B)** The numbers of copy number alterations (CNAs) with log_2_ ratios of > 0.3 or < −0.3 together with genome-wide heatmaps of probe-level intensities (log_2_ ratios) are shown. (blue: gain, red: loss).

To find genomic differences between early and invasive breast cancer lesions, pure DCIS without any invasive component from six patients, and synchronous DCIS and IDC from five patients were analyzed (Table 1). Mean coverage of the sequencing depth was 72X for both the tumor and the normal genomes. A total of 1,130 somatic mutations (1,007 point mutations and 123 indels (Table S1)) were identified in the 16 lesions (29–137 somatic mutations (median of 50.5) per lesion). We categorized the breast lesions into three groups: pure DCIS, synchronous DCIS and synchronous IDC and identified a median of 36.5 (range, 29–58), 82 (range, 37–137) and 110 mutations (range, 33–134) in each, respectively (Figure 1A). None of the mutation numbers, subtypes or spectra was significantly different between the three groups (Figure S1A–S1D, Table S2), but we observed a trend towards synchronous DCIS and IDC harboring more mutations than pure DCIS (*p* = 0.065). Consistent with previous data in breast cancer [10, 11], the C/G to T/A transition was the most common type across the cases, making up about 50% of the entire mutation (Figure S1C–S1D).

**Table 1:** Clinical and histologic characteristics of the breast tumor lesions

Case No.	Age	Diagnosis (tumor content)	TMN	Meno-pause	ER	PR	HER2 (IHC)	HER2 amplification (a-CGH)	Ki-67*	DCIS component			
										Histology	Nuclear grade	Necrosis	Size (cm)
ID1	46	IDC (85%) with DCIS (90%)	T2N0M0	Pre	+	+	+	No	Intermediate	Cribriform	High	+	3.0
ID3	58	IDC (90%) with DCIS (90%)	T2N0M0	Post	+	−	−	No	Intermediate	Cribriform	Intermediate	+	2.5
ID4	52	IDC (90%) with DCIS (90%)	T2N0M0	Post	−	−	+	Yes	High	Cribriform	Intermediate	+	3.7
ID6	50	IDC (85%) with DCIS (85%)	T2N0M0	Pre	−	−	+	Yes	Intermediate	Cribriform	High	+	2.5
ID12	47	IDC (85%) with DCIS (90%)	T2N0M0	Post	−	+	+	Yes	High	Solid	High	+	2.1
PD17	50	Pure DCIS (90%)	TisN0M0	Pre	+	+	+	Yes	Intermediate	Solid	High	+	2.0
PD18	43	Pure DCIS (90%)	TisN0M0	Pre	+	+	−	No	Low	Cribriform	Intermediate	+	3.2
PD19	43	Pure DCIS (85%)	TisN0M0	Pre	+	+	−	No	Low	Microacinar	Intermediate	+	8.1
PD21	44	Pure DCIS (85%)	TisN0M0	Pre	+	+	+	Yes	Low	Cribriform, micropapillary	High	+	5.2
PD22	52	Pure DCIS (90%)	TisN0M0	Post	+	+	−	No	Low	Cribriform	Low	−	3.9
PD23	41	Pure DCIS (90%)	TisN0M0	Pre	+	+	−	No	Low	Cribriform, solid	Intermediate	+	6.5

DCIS: ductal carcinoma *in situ*, IDC: invasive ductal carcinoma, ER: estrogen receptor, PR: progesterone receptor, TNM: tumor, lymph node and metastasis, IHC: immunohistochemistry, a-CGH: array comparative genomic hybridization.

* < 10%: Low; 10 – 30%: Intermediate; > 30%: High

### Copy number alteration profiles

a-CGH identified a total of 941 CNAs (508 gains and 433 losses, Table S3) from the 16 samples with a median of 28 (range, 8–78) for pure DCIS, 71 (range, 24–147) for synchronous DCIS and 56 (range, 25–179) for synchronous IDC (Figure 1B). There was no significant difference in the numbers of CNAs among the three groups (*p* = 0.183). However, when focusing on recurrent CNAs (≥ 3 in each group), we observed that the recurrent CNAs in pure DCIS (*n* = 42) were significantly lower than those in either synchronous DCIS (*n* = 61) or IDC (*n* = 61) (*p* = 0.041, Table S4). At an individual gene level, gains of *PIK3CA, CDK12, MLF1, EVI1, SOX2, TFRC, ERG* and *MTCP1*, and losses of *PIK3R1, APC, FGFR2, PDGFRB, CD74, ITK, EBF1, RANBP17, TLX3, NPM1, NR4A3, IL6ST* and *MAP2K4* were more frequent in synchronous DCIS or IDC than those in pure DCIS. Many of these CNAs have been identified as cancer-related with possible contributions to the development of diverse cancers [12, 13].

In the copy number profiles, we observed a total of 18 candidate chromothripses (five in pure DCIS, seven in synchronous DCIS and six in synchronous IDC) (Table S5). There was no significant difference in number of chromothripses between the three groups. The chromothripses occurred most frequently on chromosomes 8, 17 and 21 (four events each). Amplified segments in the chromothripsis areas on chromosomes 8 and 17 encompassed the *MYC* and *ERBB2* oncogenes, respectively (Figure S2).

### Genomic similarities of synchronous DCIS and IDC

**Figure 2:**
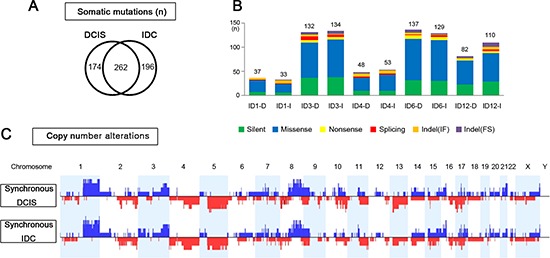
Genomic similarities of synchronous DCIS and IDC **(A)** Overlapping somatic mutations between synchronous DCIS and IDC that share 243 identical somatic variants. **(B)** Comparison of numbers and categories of somatic mutations between synchronous DCIS and IDC. **(C)** Net frequency plots of copy number alterations across whole chromosomes for the synchronous DCIS (*n* = 5) and IDC (*n* = 5).

**Figure 3:**
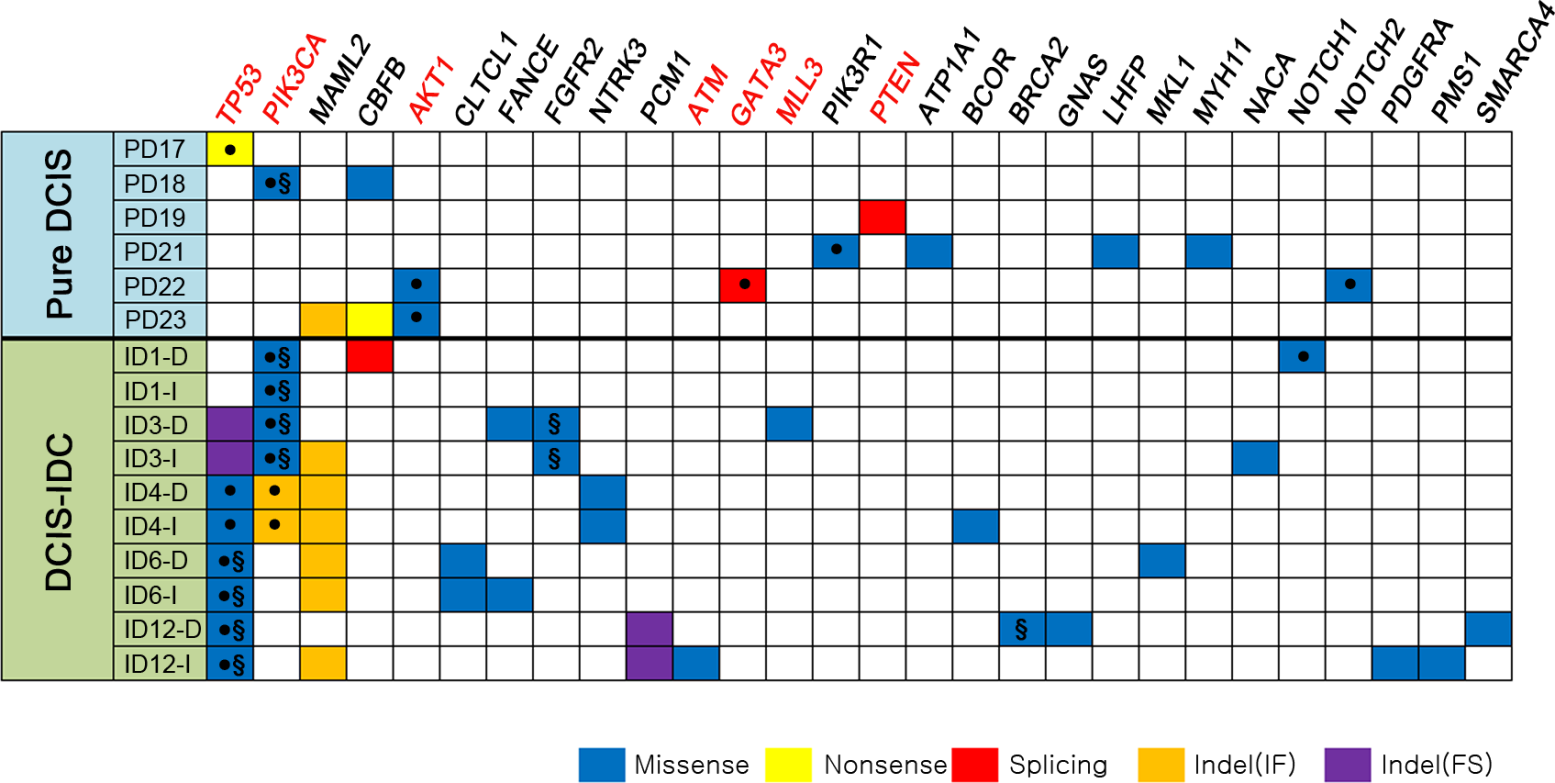
Non-silent somatic mutations in 16 breast samples referenced in the cancer Gene Census Genes with somatic mutations are listed in the order of frequencies (from left to right). The COSMIC breast cancer top 20 genes (*TP53, PIK3CA, AKT1, ATM, GATA3, MLL3* and *PTEN*) are marked in red bold. ●: The same variants have been reported in the COSMIC database, §: Suggested drivers by the CHASM analysis.

**Figure 4:**
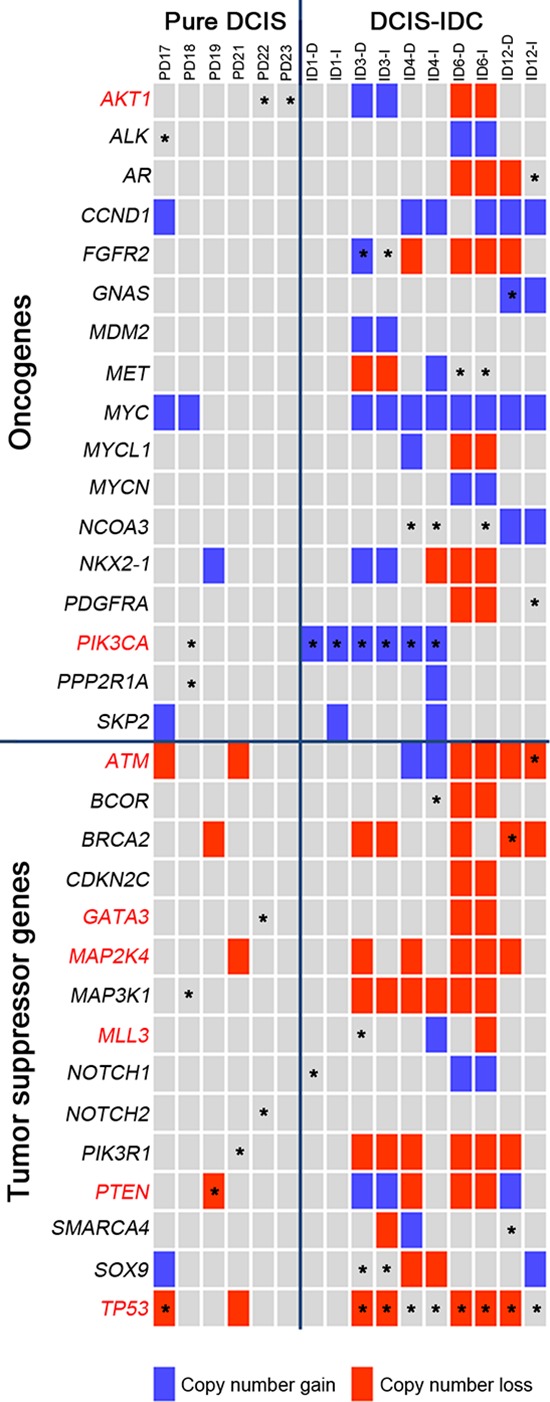
Classification of the somatic mutations and CNAs with respect to the cancer-related functions in the pure DCIS and synchronous DCIS-IDC The COSMIC breast top 20 genes are marked in red letters. Block colors represent the copy number alterations (blue: gain, red: loss). Asterisks represent the somatic mutations.

Matched DCIS and IDC (synchronous DCIS and IDC) samples showed remarkably similar patterns in both somatic mutations and CNAs in many aspects (Figure 2). Average concordance rate of the mutations between synchronous DCIS and IDC was 53.8% (range, 19.8% – 82.0%), which was far higher than the inter-IDC concordance rate (average 0.6%) or inter-DCIS concordance rate (average 0.1%) (Figure 2A–2B, Table S1). More importantly, concordance rates for both *TP53* and *PIK3CA* mutations, the most well-known mutation in breast cancers, between synchronous DCIS and IDC were 100% (Figure 3). For the CNAs, the average concordance rate was 76.6% (range, 46.2–93.1%) (Figure 2C), which was far higher than the inter-IDC (average 19.4%) or inter-DCIS concordance (average 18.5%) (Table S3). Of note, gains of *AKT1, MYC* and *PIK3CA* were present in both synchronous DCIS and IDC. In contrast, the gain of *MET*, and losses of *PTEN, BRCA2* and *TP53* were present in either one of the synchronous DCIS or IDC (Figure 4). All the 13 chromothripses in synchronous DCIS and IDC occurred in a pairwise fashion except one that occurred only in synchronous DCIS (case ID12-D) (Table S5). Since synchronous DCIS and IDC showed a high concordance, we grouped them together and termed them DCIS-IDC for comparison with pure DCIS samples.

### Cancer-related genes

To address whether the mutations found in our study could be causally implicated in the progression of DCIS to invasive disease, we queried the cancer Gene Census, a set of 483 curated cancer-related genes [14]. Overall, 28 genes with non-silent mutations in the present study were also identified in the cancer Gene Census (Figure 3). In addition, seven genes with mutations in our study overlapped with the top 20 breast cancer genes in the COSMIC database (http://cancer.sanger.ac.uk/cosmic) (Figures 3–4). Of note, there was a statistical difference in the number of potential driver genes (the cancer Gene Census) between pure DCIS (*n* = 17) and DCIS-IDC (*n* = 51) (*p* = 0.016, Table 2). At an individual gene level, 16 genes (*FGFR2, BRCA2, ATM, MLL3, GNAS, NOTCH1, PDGFRA, SMARCA4, NTRK3, PCM1, CLTCL1, FANCE, BCOR, MKL1, NACA* and *PMS1*) in the cancer Gene Census were exclusively observed in DCIS-IDC (1–7 genes per case), but not in pure DCIS (Figure 3). Interestingly, however, even the pure DCIS harbored at least one or more gene mutations in the cancer Gene Census, including *TP53, PIK3CA, AKT1, GATA3, PIK3R1* and *PTEN* (Figure 3). Genes commonly mutated in both pure DCIS and DCIS-IDC included *TP53, PIK3CA*, *CBFB* and *MAML2* (Figure 3).

**Table 2:** Summary of comparison data between pure DCIS and DCIS-IDC genomes

	Pure DCIS vs. DCIS-IDC
Number of CNAs	No significant difference
Somatic mutation numbers (Total)	No significant difference
Driver mutation numbers	Pure DCIS < DCIS-IDC (*p* = 0.022)
Mutation numbers in the cancer Gene Census	Pure DCIS < DCIS-IDC (*p* = 0.016)
Mutation numbers co-occurring with CNAs	Pure DCIS < DCIS-IDC (*p* = 0.003)
Oncogene	
Mutation numbers	No significant difference
CNA numbers	Pure DCIS < DCIS-IDC (*p* = 0.002)
Tumor suppressor gene	
Mutation numbers	No significant difference
CNA numbers	Pure DCIS < DCIS-IDC (*p* = 0.031)
Mutation numbers co-occuring with CNAs in oncogenes and tumor suppressor genes	Pure DCIS < DCIS-IDC (*p* = 0.011)

CNA: copy number alteration, DCIS: ductal carcinoma *in situ*, IDC: invasive ductal carcinoma

In addition, we performed CHASM analysis [15] to predict driver mutations. The number of predicted driver mutations in DCIS-IDC (*n* = 14) was significantly higher than that in pure DCIS (*n* = 2) (*p* = 0.022) (Table S6). Five candidate driver mutations (*BRCA2*, *FGFR2, EPHA1, DCLK3* and *PTPRB*) were detected only in the DCIS-IDC, but not in the pure DCIS (Table S6). To investigate the pathway-level relationships of the individual mutations, we performed a DAVID analysis (http://david.abcc.ncifcrf.gov) and found that mutated genes in the DCIS-IDC were significantly associated with categories of ‘notch signaling pathway’, ‘cell adhesion’, ‘cell division’, ‘DNA damage response’ and ‘p53 signaling pathway’, while pure DCIS were associated with the ‘mTOR signaling pathway’ and ‘apoptosis’ (Table S7).

### Mutation and CNA co-occurrence

To elucidate the potential synergism of mutations and CNAs of the same genes, we analyzed their co-occurrence and found that 372 mutations co-occurred with CNAs in the same samples (Table S8). DCIS-IDC harbored significantly more co-occurrences (*n* = 344) than pure DCIS (*n* = 28) (*p* = 0.003). Among them, *PIK3CA, TP53, FGFR2, BRCA2, ATM, CBFB, GNAS, LHFP, MAML2* and *WHSC1* genes were listed in the cancer Gene Census as well. When displaying somatic mutations and CNAs together with respect to function (oncogenes or tumor suppressor genes [16]) (Figure 4), we found that oncogenes *PIK3CA, FGFR2* and *GNAS* involved both somatic mutations and copy number gains and that tumor suppressor genes *TP53, PTEN, BRCA2* and *ATM* involved both somatic mutations and copy number losses. Such co-occurring events with functional correlation were significantly higher in DCIS-IDC than in pure DCIS (*p* = 0.011, Table 2).

### Higher genomic alterations in progesterone receptor-negative breast cancers

**Figure 5:**
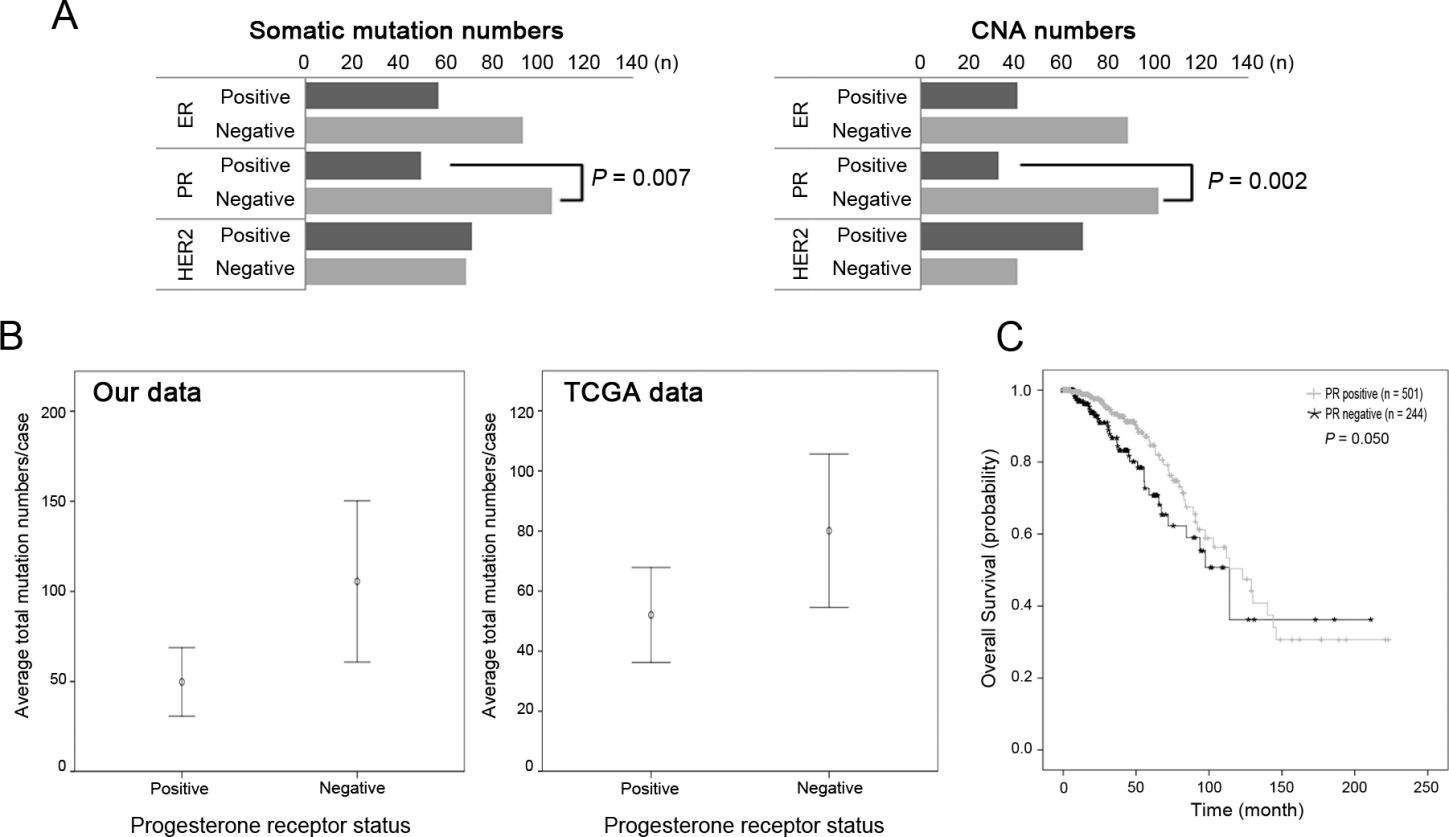
Somatic mutations and copy number alterations according to the receptor status **(A)** The PR-negative group harbored significantly more mutations and copy number alterations (CNAs) than the PR-positive group (*p* = 0.007 and *p* = 0.002, respectively). **(B)** Similar distribution of the higher mutation numbers in PR-negative group in the TCGA data (*p* = 1.47 × 10^−17^). **(C)** Survival analysis of PR-positive and PR-negative group in the TCGA data. PR-negative group showed worse prognosis than PR-positive group (*p* = 0.050).

Finally, we queried genomic alterations with respect to clinicopathologic features (Table 1). Only progesterone receptor (PR) was significantly associated with genomic alteration profiles. PR (−) tumors were associated with a great number of somatic mutations (*p* = 0.007), CNAs (*p* = 0.002), co-occurrence of mutation/CNAs (*p* = 0.005) and the cancer Gene Census (*p* = 0.003) (Figure 5A, Table S9). This finding was in agreement with public data from TCGA, which also showed that the PR (−) group harbored more mutations and worse prognosis than the PR (+) group (Figure 5B–5C).

## DISCUSSION

Although considerable genomic data has been produced for advanced breast cancer lesions (mainly IDC), whole-exome sequencing has rarely been applied to early lesions (mainly DCIS). The aim of our study was twofold. First, we attempted to identify somatic mutations and genome-wide CNAs for both pure DCIS and synchronous DCIS with IDC. Second, we attempted to detect genomic differences between DCIS and IDC that might drive DCIS to progress to IDC. We found that genomic alterations for pure DCIS were comparable to those for synchronous DCIS-IDC in quantity (i.e., total mutation and CNA numbers), but that driver alterations for pure DCIS were less common than those for synchronous DCIS-IDC (i.e., numbers of driver mutation and co-occurrence of mutation/CNAs). Our data indicate that pure DCIS may have qualitatively less aggressive genomes that may need further driver hits to develop into IDC genomes.

To find critical determinants for DCIS progression to IDC, we utilized the CHASM analysis for driver gene identification and found that synchronous DCIS-IDC harbored many more drivers than pure DCIS. However, we could not pinpoint recurrent determinants for the progression. These data indicate that there may be neither a single driver nor a recurrent group of drivers for the progression, but that non-recurrent drivers might cooperate together to encourage progression. Somatic mutations of *FGFR2, BRCA2, MET, SMARCA4, AR, GNAS, NCOA3, PDGFRA, ATM, BCOR, MLL3, NOTCH1* and *SOX9,* and CNAs in *AKT1, ALK, FGFR2, GNAS, MDM2, MET, MYCL1, MYCN, NCOA3, FGFR2* (gains), *BCOR, CDKN2C, GNAS, GATA3, MAP3K1, NOTCH2, PIK3R1, SMARCA4* and *SOX9* (losses) were identified as synchronous DCIS-IDC-specific alterations in our study (Figure 4) that may cooperate for progression. In our study, *FGFR2* is not only mutated but also harbors a copy gain. FGFR2 interacts with fibroblast growth factors, setting in motion a cascade of downstream signals, ultimately influencing mitogenesis and differentiation [17]. Somatic mutations of *FGFR2* have been reported in many cancers, including breast cancers [18] and *FGFR2* gene variations confer a risk for breast cancer [19]. DCIS-IDC harbored significantly different CNAs at 11q13.4, 17q12 and 17q22 compared to pure DCIS, a finding in agreement with previous studies [6] that strongly suggests a role for these loci in progression.

Despite the lower prevalence of driver mutations in pure DCIS than synchronous DCIS-IDC, even pure DCIS with a low nuclear grade (case PD22) harbored at least one driver such as *TP53, PIK3CA*, *AKT1, PTEN, GATA3* and *PIK3R1* mutations, suggesting that these drivers may be essential for the early phase of DCIS development and that gradual accumulation of driver mutations might be required for progression. Some genes displayed alterations in both pure DCIS and DCIS-IDC, indicating their roles in both initiation and progression/maintenance of breast cancers. For example, the most common mutation in our study was *TP53*, which was more prevalent in DCIS-IDC (4/5) than pure DCIS (1/6) (*p* = 0.042). However, this difference might result from selection bias, as previous data did not show a significant difference [16]. The second most common mutation was *PIK3CA* [20, 21]. Interestingly, all *PIK3CA* mutations in DCIS-IDC co-occurred with copy number gains, whereas *PIK3CA* in pure DCIS did not (Figure 4). PIK3CA signaling could be activated by other gene alterations such as *AKT1* and *PTEN* [28]. The majority of cases in both pure DCIS (4/6) and synchronous DCIS-IDC (4/5) harbored at least one of these three alterations (*PIK3CA, AKT1* and *PTEN*) in our study as identified previously [9, 10, 22].

Chromothripsis has been observed across many cancer types, including IDC [23], but has not been evaluated before in DCIS. A prevailing view has supported early occurrence of chromothripsis during cancer evolution [23], but ‘how early’ has been undefined. We found chromothripsis events in pure DCIS as well as synchronous DCIS-IDC, indicating that it may occur early in breast cancer development and might play a role in the initiation phase of breast cancer.

The steroid hormones, estrogen and progesterone, are critically linked to breast cancer development [24]. Hormone receptor status in breast cancer is important in prognosis (poor in triple-negative cancers) and therapeutic applicability (tamoxifen treatment for ER (+)). Genomic alterations are not always sufficient to drive breast cancer development but additional factors such as hormonal environment may contribute to development and progression [24]. We discovered that not only mutation numbers, but also other genomic parameters such as CNAs, co-occurrence of mutation/CNAs and driver genes were correlated with PR-negativity. In addition, TCGA data show that PR (−) breast cancers had worse prognosis than PR (+) cases. A previous large population cohort study found that PR-negativity was an independent poor prognostic variable in all four subgroups of breast cancers [25]. The expression of PR is directly related to estrogen binding to ER and the function of PR is dependent on the normal structure and function of ER [26], which would account for relative unresponsiveness to endocrine therapy in PR (−) breast cancers [25]. However, such a connection between ER and PR does not fully explain the poor prognosis of PR (−) breast cancer patients [25]. In this study, we found evidence that genomic aggressiveness in PR (−) breast cancers could be an underlying factor for poor prognosis.

Previously, there have been similar studies to our study describing the differences between DCIS and IDC at genomic level [6, 7]. Regarding the sample size, previous studies (13 paired DCIS/IDC cases in one report, and 16 cases of pure DCIS and 6 paired DCIS/IDC in the other study) analyzed more cases than ours (6 cases of pure DCIS and 5 paired DCIS/IDC cases). Despite the smaller cases analyzed, our study may have several advantages over the previous studies to get more comprehensive understanding about the genomic aberrations that may contribute to the progression of DCIS to invasive diseases. First, we adopted whole-exome sequencing, which had not been used in the two studies [6, 7]. Second, the array-CGH platform used in this study (180K oligoarray) could provide more accurate and reliable CNA data compared with the array-CGH platforms used in the previous studies (19 K cDNA array and 32 K BAC array). Third, to guarantee reliable mutation detection, we used fresh frozen tissues whereas a previous study [7] used formalin-fixed paraffin-embedded tissues. The limited availability of fresh frozen tissue was the reason why we were not able to expand the sample size.

**Figure 6:**
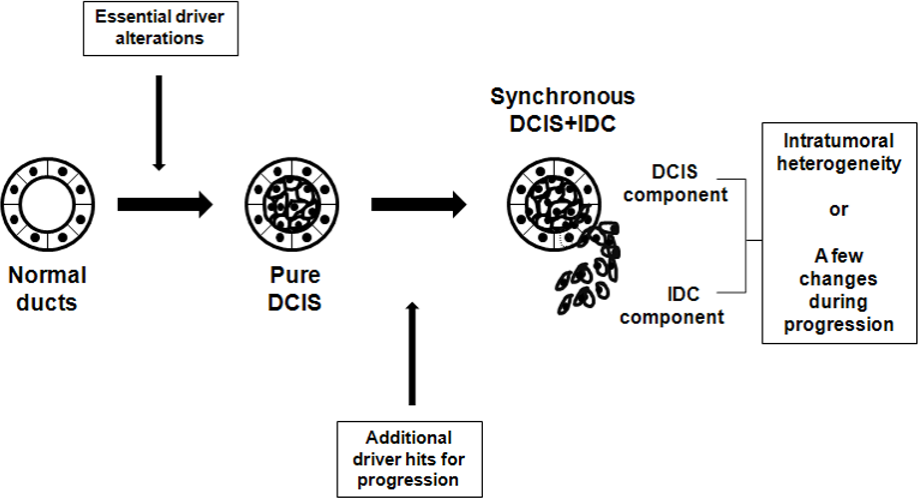
Schematic representation of suggested genomic status of pure DCIS, synchronous DCIS and IDC Development of pure DCIS requires essential genetic driver alterations, to which more genetic alterations are added for progression to synchronous DCIS-IDC. No significant genomic difference between IDC and synchronous DCIS suggest that they are genetically at the same stage with just intratumoral heterogeneity or minimal genetic changes during progression to IDC.

In summary, pure DCIS is a neoplastic lesion that already harbors some driver alterations, but needs more drivers to become an invasive disease (Figure 6). Such early fixation of some driver mutations provides rationale for careful clinical management of pure DCIS. Our findings also indicate that neither a single gene nor a recurrent group of genes determines whether pure DCIS cells progress to IDC. We also found that the genomic features of DCIS associated with IDC were closer to IDC than pure DCIS. No significant genomic difference between IDC and synchronous DCIS suggest a possibility that these two histologically distinct lesions are genetically at the same stage, but show just intratumoral genetic heterogeneity. Another possibility is that during progression to IDC there are subtle genetic changes that may not be easily differentiated (Figure 6). Both possibilities suggest that even a histologically early lesion (DCIS) associated with IDC should be considered a possibly invasive lesion at the genomic level. By looking at all the evidence together, it might be possible to determine whether newly found DCIS after surgery is a residual tumor or newly developed pure DCIS. Finally, the association of PR-negativity and increased genomic burden may provide clues for further subclassification of breast cancers, enhancing diagnosis and management.

## MATERIALS AND METHODS

### Breast tumor tissues

IDC tissues simultaneously resected with adjacent DCIS (synchronous DCIS with IDC) from five patients and pure DCIS tissues from six patients were obtained from the Tissue Bank at Seoul St. Mary Hospital (Seoul, Korea). All patients except one Russian woman were Korean and had no family history of breast cancer. None of the patients had tumor recurrence and were disease-free for up to five years after surgery. Approval for this study was obtained from the institutional review board of the Catholic University of Korea, College of Medicine. Clinicopathologic features of the patients are summarized in Table 1. Synchronous DCIS with IDC patients seemed older, more postmenopausal and to have higher proliferation rates (Ki-67) than the pure DCIS patients. These features may reflect the natural history of less aggressive DCIS progression to IDC with a long latency. Initially, frozen tissues from the tissue bank were cut, stained with hematoxylin/eosin and examined under a microscope by a pathologist. The frozen tissues selected for the study were serially cut and lightly stained with hematoxylin without fixation (Figure S3). IDC and DCIS cells were selectively procured from frozen sections using a 30G1/2 hypodermic needle by microdissection as described previously [27]. IDC and DCIS cell purities from the microdissection were approximately 85 – 90%. To minimize DNA degradation, we finished the processes from cutting to microdissection within 120 minutes. For normal DNA, we used frozen tissue from matched patients devoid of IDC and DCIS. For genomic DNA extraction, we used the DNeasy Blood & Tissue Kit (Qiagen, Hilden, Germany) according to the manufacturer's instructions.

### Whole-exome sequencing

DNA from tumor tissue (6 cases of pure DCIS, 5 synchronous DCIS and 5 synchronous IDC) was separately analyzed for whole-exome sequencing using the Agilent SureSelect Human All Exome 50 Mb Kit (Agilent Technologies, Santa Clara, CA) according to the manufacturer's instructions. All samples were matched with normal genomes to identify somatic mutations. Using the Illumina HiSeq2000 platform to generate 101 bp paired-end reads, the Burrows-Wheeler aligner was used to align the sequencing reads onto the human reference genome (hg19). The aligned sequencing reads were evaluated using Qualimap [28]. Detailed information about the sequencing alignments is shown in Table S10. Somatic variants were identified using MuTect [29] and SomaticIndelDetector [30] for point mutations and indels, respectively. The ANNOVAR package was used to select somatic variants located in the exonic sequences and predict their functional consequences [31].

### DNA copy number profiling

DNA copy number profiling was performed using the Agilent Sure Print G3 Human comparative genomic hybridization (CGH) Microarray 180 K. The genomic DNA of breast tumor tissues and matched normal genomes was hybridized onto the array according to the manufacturer's instructions. Background correction and normalization for array images was performed using Agilent Feature Extraction Software v10.7.3.1. The RankSegmentation statistical algorithm in NEXUS software v7.5 (Biodiscovery Inc., El Segundo, CA) was used to define the CNAs of each sample; a log2 ratio larger than 0.3 was identified as gain and lower than −0.3 as loss. The a-CGH results from patients 3 and 12 were of poor quality and deemed inappropriate for analysis, so the copy number alterations for these samples were generated from whole-exome sequencing data. The inference of chromothripsis was manually curated by examining cases with > 10 identifiable shifts in the copy number profiles per chromosome.

### Driver mutation and gene set analyses

To discover candidate driver gene mutations contributing to tumor development and progression, the CHASM analysis program was used with the ‘breast’ category for cancer tissue type [15]. FDR ≤ 0.3 was identified as a criterion for driver mutations. To investigate the gene ontology of the mutations of each grouped sample, we performed DAVID analysis (http://david.abcc.ncifcrf.gov/) [32]. Three categories (‘biological process’, ‘cellular components’, ‘molecular function’) and 'KEGG pathway’ were identified and sorted by significance. Detailed information is shown in Table S7.

## SUPPLEMENTARY MATERIAL ON THE INTERNET
















