# Supplementary material for: Genomic differences between pure ductal carcinoma in situ and synchronous ductal carcinoma in situ with invasive breast cancer
Source: Oncotarget. 2015 Mar 26;6(10):7597–607. doi: 10.18632/oncotarget.3162 (PMC4480702; doi:10.18632/oncotarget.3162)
Supplement: Supplementary file 1 [file oncotarget-06-7597-s001.pdf]

SUPPLEMENTARY MATERIAL ON THE INTERNET

The following supplementary material may be found in the online version of this article:

SUPPLEMENTARY FIGURES AND TABLES

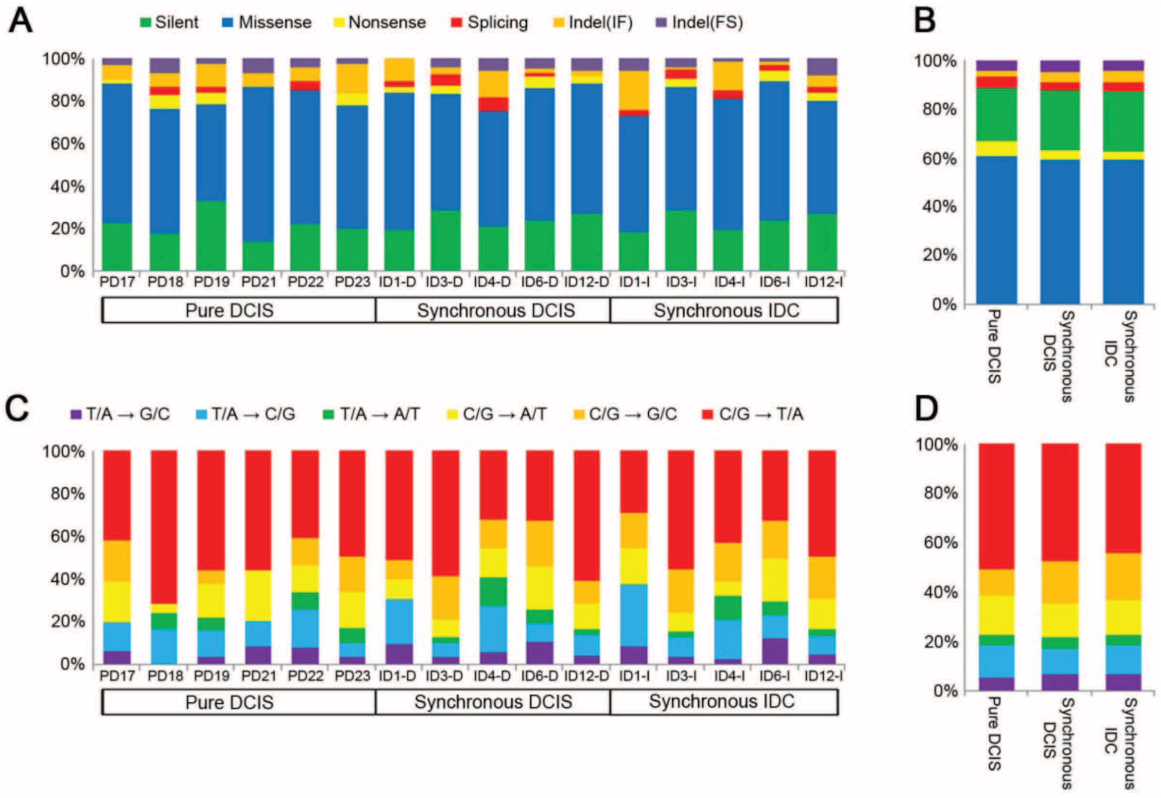

Supplementary Figure S1: Analysis of mutation types and spectra across the breast tumors. (A) Relative fractions of mutation types of each sample are shown. **(B)** Relative fractions of mutation types in pure DCIS, synchronous DCIS, and synchronous IDC are shown. There is no significant difference among them. **(C)** Relative fractions of mutation spectra in each sample are shown. **(D)** There is no significant difference of the mutation spectra among them.

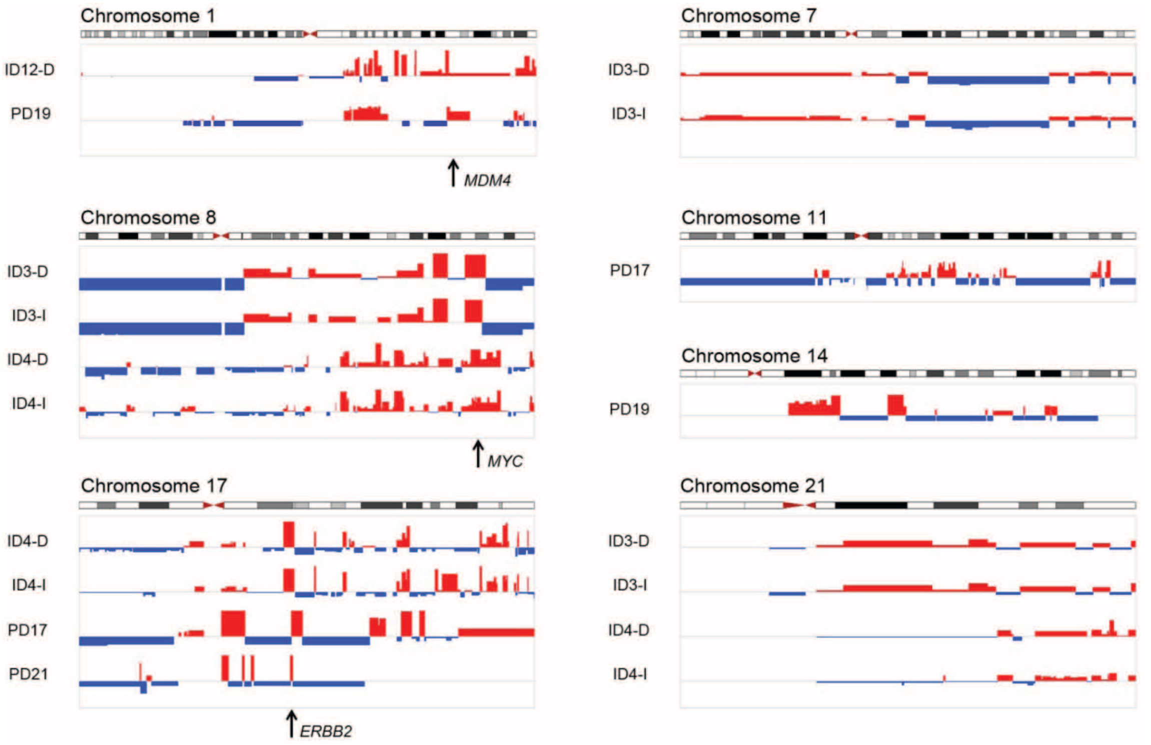

Supplementary Figure S2: Chromothripsis events in breast cancer genomes. A total of 18 candidate chromothripsis events on chromosome 1, 7, 8, 11, 14, 17, and 21 were identified in six breast cancers. The red and blue bars represent the $\log_2$ signal intensities for copy gain and loss segments, respectively.

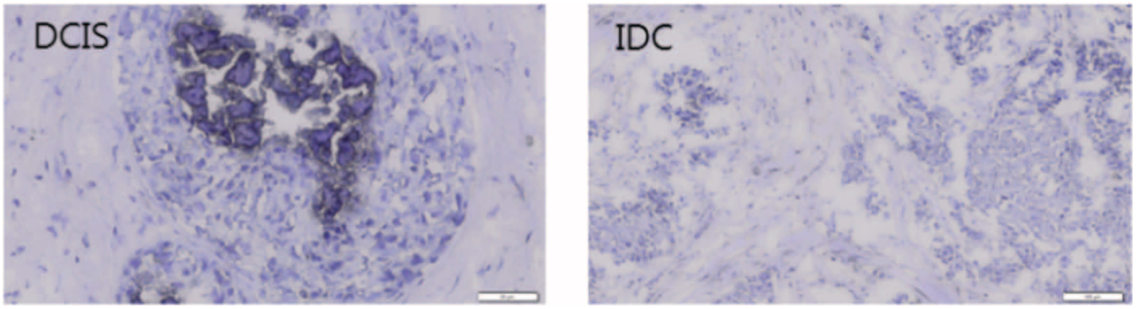

Supplementary Figure S3: Histology of frozen sections used for microdissection. Frozen tissues of DCIS (left) and IDC (right) were cut and stained with hematoxylin without treatment of any fixatives. Tumor cells microdissected each from DCIS and IDC were used for whole-exome sequencing and a-CGH analyses.

Supplementary Table S1: Somatic mutations identified by whole-exome sequencing in breast tumor samples

Supplementary Table S2: Mutation and copy number alteration profiles across the samples analyzed. There is no significant difference in mutation type, mutation spectra, and CNAs among the three groups of pure DCIS, synchronous DCIS, and synchronous IDC. There is a trend of synchronous DCIS and IDC harboring more mutations than pure DCIS.

Supplementary Table S3: Copy number alterations of pure DCIS, synchronous DCIS, and synchronous IDC assessed by array-comparative genomic hybridization.

Supplementary Table S4: Recurrent copy number alteration (CNA) regions. CNAs identified in more than three patients were considered as recurrent. CNAs identified in more than three patients were considered as recurrent.

Supplementary Table S5: Chromosomal locations of chromothripsis events in the breast tumors.

Sample_ID	Type	Chromosome																						
		1	2	3	4	5	6	7	8	9	10	11	12	13	14	15	16	17	18	19	20	21	22	X
ID1	Synchronous DCIS																							
	Synchronous IDC																							
ID3	Synchronous DCIS							O	O													O		
	Synchronous IDC							O	O													O		
ID4	Synchronous DCIS								O									O				O		
	Synchronous IDC								O									O				O		
ID6	Synchronous DCIS																							
	Synchronous IDC																							
ID12	Synchronous DCIS	O																						
	Synchronous IDC																							
PD17	Pure DCIS											O						O						
PD18	Pure DCIS																							
PD19	Pure DCIS	O													O									
PD21	Pure DCIS																	O						
PD22	Pure DCIS																							
PD23	Pure DCIS																							

Supplementary Table S6: Suggested driver mutations by CHASM analysis. All mutations identified across the breast samples were analyzed by CHASM program using 'breast' as the tissue type. The mutations with the FDR ≤ 0.3 were considered as 'drivers'

Supplementary Table S7: Gene ontology analysis of mutations using DAVID. The mutations categorized as pure DCIS and synchronous DCIS-IDC were analyzed by DAVID analysis (http://david.abcc.ncifcrf.gov/) and listed according to significance.

Supplementary Table S8: List of mutations which co-occurred with copy number alterations

Supplementary Table S9: Genomic differences according to the hormone receptor status

	ER*			PR			HER2		
	Positive (*n* = 10)	Negative (*n* = 6)	*P*-value**	Positive (*n* = 10)	Negative (*n* = 6)	*P*-value	Positive (*n* = 10)	Negative (*n* = 6)	*P*-value
Somatic mutations	571	559	0.056	497	633	0.007	716	414	0.713
CNAs	411	530	0.118	332	609	0.002	692	249	0.263
Mutation co-occur with CNAs	158	214	0.031	88	284	0.005	242	130	0.263
COSMIC overlap	34	34	0.022	33	35	0.007	44	24	0.713
Driver mutations	8	8	0.368	9	7	0.492	10	6	0.958

*ER: estrogen receptor; PR: progesterone receptor; HER2: human epidermal growth factor receptor 2
***P*-values in red indicate the statistical significance.

Supplementary Table S10: The description of whole-exome sequencing data

Samples*	Sequencing reads	Mapped (%)	Coverage (mean)	% of >20 bases
ID1-N	56,898,089	55,673,615 (97.85%)	71.35	84.7
ID1-D	60,636,059	59,331,140 (97.85%)	76.34	84.6
ID1-I	58,385,854	56,934,861 (97.51%)	75.68	85.1
ID3-N	53,583,322	52,456,316 (97.90%)	68.67	83.4
ID3-D	51,751,133	50,598,818 (97.77%)	64.87	79.6
ID3-I	52,830,131	51,608,148 (97.69%)	67.86	80.8
ID4-N	47,902,111	46,829,044 (97.76%)	60.75	80.7
ID4-D	55,167,932	54,054,369 (97.98%)	69.99	82.0
ID4-I	55,279,391	54,103,473 (97.87%)	69.35	82.9
ID6-N	54,217,958	53,068,244 (97.88%)	68.73	83.3
ID6-D	53,488,761	52,195,794 (97.58%)	67.42	80.1
ID6-I	57,318,602	56,062,789 (97.81%)	73.55	83.3
ID12-N	68,128,707	66,506,817 (97.62%)	85.29	87.0
ID12-D	58,098,612	56,770,814 (97.71%)	71.82	83.0
ID120T	52,957,734	51,764,081 (97.75%)	66.94	82.8
PD17-N	68,043,358	67,054,064 (98.55%)	82.27	84.6
PD17-D	58,379,747	57,733,451 (98.89%)	72.71	81.1
PD18-N	55,989,276	55,280,558 (98.73%)	71.44	83.6
PD18-D	58,412,934	57,836,684 (99.01%)	76.30	84.3
PD19-N	55,703,985	54,933,008 (98.62%)	71.09	83.0
PD19-D	59,564,061	58,460,371 (98.15%)	76.61	83.9
PD21-N	58,156,123	57,389,151 (98.68%)	72.68	82.6
PD21-D	54,984,748	54,321,935 (98.79%)	69.55	81.4
PD22-N	55,231,054	54,474,346 (98.63%)	68.11	82.0
PD22-D	50,826,624	50,235,113 (98.84%)	66.71	80.9
PD23-N	59,931,521	59,067,403 (98.56%)	73.39	83.0
PD23-D	61,238,112	60,491,176 (98.78%)	78.91	84.2

*The tumor and the matched normal genomes are discriminated as ‘N’ for normal, ‘D’ for ductal carcinoma *in situ*, and ‘I’ for invasive ductal carcinoma.
